# Factors Predictive of Behavioural and Emotional Dysfunction in Adolescents in a Secure Children’s Home

**DOI:** 10.1007/s40653-020-00329-7

**Published:** 2020-11-17

**Authors:** Rebecca Harris, Daniel R. Stubbings, Karen De Claire

**Affiliations:** 1 Hillside Secure Children’s Home, Neath, UK; 2 School of Sport and Health Sciences: Applied Psychology Department, Cardiff Metropolitan University, Cardiff, UK

**Keywords:** Secure hospital, Adolescents, Adverse childhood experiences, Risk

## Abstract

The aim of the present study was to investigate what factors are predictive of behavioural and emotional dysfunction in adolescence. A sample of 60 young people accommodated under a welfare or youth custody service order in a UK secure children’s home between 2016 and 2018 was used. Data regarding young people’s Adverse Childhood Experiences (ACE’s) history, scores on standard assessments and factors thought to indicate behavioural and emotional dysfunction were collected from file information. Correlations and regression models were used to analyse the data. Analyses found that young people in this setting had been exposed to more ACEs than the general population. Furthermore, signs of behavioural and emotional dysfunction as a result of exposure to ACE’s appeared to be present from early adolescence. It was found that exposure to verbal and sexual abuse were the greatest predictors of involvement in risk incidents. The young person’s substance misuse habits were the best predictor of the length of stay in the secure children’s home. These findings may have policy implications and highlight the need for early interventions with young people exposed to ACE’s.

There appears to be a relationship between the experiences a person may face when they are young and how they perceive and act in their world during adulthood (Bowlby 1969; National Research Council 2014; Raby et al. 2015; Fletcher and Schurer 2017). This relationship is present for both positive childhood experiences, such as a nurturing and supportive family environment, as well as experiences that are more adverse, such as witnessing domestic violence within the household. Whilst there is a vast amount of research investigating the impact of childhood on future outcomes, it is not clear what factors are predictive of negative outcomes in adolescence, such as involvement in severe incidents or length of time spent in a secure accommodation. Whilst it is believed that experiencing adversity during childhood may play a role, there has been insufficient research conducted in this area.

The term Adverse Childhood Experiences (ACEs) was first coined by Felitti et al. (1998). ACEs are defined as stressful or traumatic events that occur in a child’s life before the age of 18 that may have negative consequences for future development, such as poor health outcomes, for example mental illness, and negative social outcomes, including involvement in violence and unemployment. During the original ACE study, Felitti et al. (1998) identified 10 categories of ACEs. These ACEs were defined as incidents of physical, sexual or verbal abuse, physical and emotional neglect, witnessing domestic violence, living with an adult with substance misuse issues or mental health problems, living in a household where parents have separated and having a member of the household incarcerated as a child. More recent research into the effects of ACEs has expanded the definition to include incidents of peer or sibling victimisation, parental death during childhood and growing up in poverty, which have been demonstrated to have a harmful effect on a child’s development (Finkelhor et al. 2013).

Hillis et al. (2001) investigated the effects of exposure to adverse experiences during childhood on sexual behaviours in 5060 females aged 25 and older. Researchers found that as the number of ACE incidents increased, so too did the number of sexual partners women reported having and their self-perceived risk of AIDS, for example, in women who reported experiencing 6–7 ACEs, 12% reported having 30 or more partners during their lifetime. Furthermore, it was found that the risk of early onset of sexual behaviours, including intercourse, also increased amongst women with elevated numbers of adverse experiences, with 31% of women with 6–7 ACEs also reporting engagement in sexual activity from a young age, compared to 4% of woman with no ACEs. It was suggested that as a result of being exposed to adverse experiences during their childhood, the young people had grown up in families that were unable to provide them with protection and secure relationships, and therefore the risky sexual behaviours may have symbolised an attempt to fulfil a need for intimate personal connections. Goodson et al. (1997) identified a number of risk factors that are associated with engaging in sexual activity from an early age and having multiple sexual partners. These included living in poverty, using drugs and alcohol, having unsupportive parents and performing poorly at school. However, due to Hillis et al.’s (2001) participants having both been exposed to adverse experiences and engaging in risky sexual behaviours during adolescence, it is not clear whether exposure to ACEs always preceded, rather than followed, these risky behaviours.

One of the first United Kingdom (UK) studies exploring the effects of ACEs on future development was conducted by Bellis et al. (2013). The retrospective study involved 1500 participants aged 18–70 years who were asked to complete an ACE questionnaire based on their life experiences. Researchers found that at least 47% of individuals in the study reported at least one ACE. Furthermore, it was reported that as ACE scores increased, so too did the likelihood of experiencing adverse mental, behavioural and physical outcomes throughout the life course, for example, those with an ACE score of 4 or more were 14 times more likely to have been a victim of violence over the last 12 months and 20 times more likely to have been incarcerated at some point in their lifetime. Additionally, Bellis et al. (2013) also found that there was a negative relationship between a person’s ACE score and ratings on life satisfaction and mental wellbeing scales. However, as this study relied on participants recalling past life experiences, it may be possible that some participants chose not to reveal the true nature of their childhood experiences, or simply forgot, therefore, the actual prevalence of ACEs from this UK population may be much higher.

The dose-dependent effect of ACE’s has frequently been recorded in the literature, suggesting that the higher an individual’s ACE score, the more at risk they are of experiencing negative outcomes such as incarceration or health problems later in life. However, there has been relatively little research examining the effect of the type of ACE on predicting outcomes. It may be the case that, whilst the number of ACEs a person experiences is important, so too is the type of ACEs that a person has been exposed to in forecasting what outcomes they may be at risk of experiencing in the future, be it health or social outcomes. A meta-analysis conducted by Norman et al. (2012) found that those who had been exposed to emotional abuse were more at risk of developing depression compared to those who had experienced physical abuse, whilst participants who had been exposed to physical abuse had higher odds ratios for drug abuse compared to participants who had experienced emotional abuse.

The relationship between the types of ACEs a person has been exposed to and future outcomes has also been demonstrated when investigating specific types of offences committed by offenders who have a history of adversity during childhood. Reavis et al. (2013) completed the ACE questionnaire with four different offender groups (domestic violence offenders, sexual offenders, non-sexual child abusers and stalkers; *n* = 151) and a normative sample of adult males. The aim of the study was to investigate whether the presence of ACEs could be linked to antisocial behaviours, specifically, in a criminal population. Relative to the normative sample, all 4 offender groups reported experiencing approximately four times as many ACEs. Furthermore, it was also found that participants that had been convicted of child abuse or sexual offending were almost twice as likely to report experiencing sexual abuse during childhood compared to other offender groups. However, researchers in this study relied on self-reports of childhood experiences, so once again, these results should be interpreted with caution due to the potential inaccuracy of self-reported data.

It is also important to consider that being exposed to ACEs may affect a person’s risk of victimisation in the future. Ports et al. (2016) found that as an individual’s ACE score increased, so too did their risk of sexual victimisation in adulthood. Compared to those who have not experienced or witnessed violence in the household, those who have been victims of child abuse or witnesses to domestic violence during childhood are more likely to experience abuse from a partner during adulthood, particularly women (ONS 2018).

Duke et al. (2010) investigated the relationship between childhood adversity and aggressive displays of behaviour during childhood. Students from the 6th, 9th and 12th grades (*n* = 136,549) were recruited and asked to report on any adverse experiences they had been exposed to, including sexual abuse by family members, physical abuse, family drug and/or alcohol use and witnessing abuse within the household. Participants were then asked to report any history of involvement in violent behaviour, such as physical fighting, carrying a weapon and dating violence. Researchers found an increased risk of violence for every adverse event identified in males, for example, boys who had been molested by a family member during childhood were 45 times more likely to have a history of dating violence as an adolescent.

The effects of ACEs on the lives of children has also been explored by Baglivio et al. (2014). ACE prevalence in a population of 64,329 juvenile offenders was examined across genders to produce an ACE composite score and a risk to reoffend classification level, using the Positive Achievement Change Tool (PACT) risk assessment, which was compared to the results of ACE studies conducted on adults. It was found that female youths reported higher rates of ACEs compared to males, with females on average reporting exposure to at least four ACEs, whilst males on average reported three to four ACEs. Analyses also found that when compared to rates of ACEs in a study by Felitti et al. (1998), the juvenile offenders used in by Baglivio et al. were significantly more likely to have been exposed to ACEs and had a greater likelihood of being exposed to multiple ACEs. Finally, it was found that ACE exposure not only increases the likelihood of becoming involved in the juvenile justice system, it also increases the risk of re-offending. The use of self-report ACE measures in juvenile offenders may be advantageous relative to older offenders, as their memories are likely clearer given that their experiences occurred in the more recent past. This may translate to more accurate self-reports compared to older participants. Later research from Baglivio et al. (2015) found that, using a juvenile offender sample, a higher ACE score was associated with a greater likelihood of arrest during childhood and adolescence, as well as an earlier age of first arrest.

Young people that are at risk of absconding from a place of safety and are at significant risk of harming themselves or others will meet the criteria to reside in a secure children’s home. There are 14 secure children’s home within the UK that provide specialist individually tailored therapeutic treatment to vulnerable young people (securechildrenshomes.org.uk^2020^). The aim of these units is to promote skill development, reduced behaviours that challenge, address mental health issues and rehabilitate them back into the community. A rich data set is often collected in these settings as part of routine service and patient monitoring. The residents stay in the secure children’s homes for a minimum of one month and this allows for a range of assessments to be undertaken and progress documented. The need to investigate the data available from secure children’s homes is vital in order to examine the factors that lead to the young people being placed there.

The aim of the present study was to build on the limited literature base exploring the predictive factors of emotional and behavioural dysfunction in a sample of adolescents in a secure children’s home. Specifically, the present study aimed to investigate the possible relationship between ACEs and negative outcomes, such as prolonged periods of time spent in a secure children’s home and the likelihood of the young people presenting as risky to themselves or others. The analysis was conducted on a sample recruited from a secure children’s home in the UK. These settings aim to provide a safe and secure environment for young people, aged between 10 and 17 years to allow their individual needs to be supported following traumatic incidents in their past or a crime that has been committed. Whilst in residence young people are offered a range of psychological assessments and multi-agency involvement to deliver individualised care plans. The present study used routinely collected data from young people who had been accommodated in the secure children’s home between the years 2016 and 2018. This data was used in order to investigate whether there was a relationship between these variables and the number of incidents young people are involved in whilst in the secure children’s home and their length of stay. It was hypothesised that those who had been exposed to at least one ACE will have a greater likelihood of exposure to multiple ACEs. It was also expected that there will be a relationship between different types of ACEs and negative outcomes, such as behaviour dysfunction in childhood. That is, as the young people’s ACE scores increase, so too will the number of incidents that young person is involved in, as well as their length of stay.

## Methods

### Participants

The data used in this study was collected from file information of 77 young people (aged 14–18 years) who had resided at the Hillside Secure Children’s Home at any time between 2015 and 2018. Of these young people, 57 were males and 20 were female. The young people had been accommodated at the secure children’s home under either a welfare order (*n* = 52), where they were deemed to be a danger to themselves or other people, or under a youth custody service order (YSC; *n* = 25), where the young people had committed a crime but were thought to be too vulnerable to be placed in a youth offender’s institution.

### Measures

#### ACE Score and Other Adverse Experiences

Adverse Childhood experience include three domains; abuse (physical, emotional/verbal and sexual), neglect (physical and emotional), and household dysfunction (mental illness, parental incarceration, domestic violence, parental substance abuse and parental separation) (Felitti et al. 1998). In the our data set there was insufficient information to determine whether the young people had faced emotional or physical neglect therefore the overarching category of ‘neglect’ was used. Young people’s ACE scores were calculated by reading through available file information and marking each ACE as either present or not present in the child’s history. There are no cut-offs scores or levels of severity noted as part of this procedure.

Information related to other potentially relevant behaviours was also collected. The presence of substance misuse problems was noted in the case history. If the child was exposed to sexual exploitation, defined as the child being given money or goods in exchange for sexual acts, had also been documented. These acts often involved manipulation or violent coercion. The category of child sexual exploitation also included instances of human trafficking in some cases.

#### Trauma Symptom Checklist for Children (TSCC)

The TSCC (Briere 1996) was designed to measure the severity of post-traumatic stress and related psychological symptomology in children who have been exposed to, or experienced traumatic events. The TSCC covers domains such as anxiety, PTSD and anger, with scores of 65 or above being deemed as a clinically significant presence. The TSCC also contains three scales regarding sexual concerns where a score of 70 or above is deemed clinically significant. The TSCC has been demonstrated to have both internal consistency and test-retest reliability and sound validity in both clinical and non-clinical samples.

#### WASI-II

The Wechsler Abbreviated Scale of Intelligence (WASI-II) (Wechsler 2011) provides a brief measure of cognitive ability and is used across the ages of 6–90. The measure is comprised of four subtests. The block design and matrix reasons are used to comprise the performance intelligent quotient (IQ), which gives an indication of the individuals non-verbal fluid abilities and visuomotor/co-ordination skills. The vocabulary and similarities subtests are used to comprise the verbal IQ, which gives an indication of an individuals crystallised abilities. The full-scale IQ provides an indication of global functioning at the time of assessment. Full-scale IQ scores corresponds to the following ranges; below 70 is low functioning occurring in 2.2% of the population, 70–80 is borderline intelligence occurring in 6.7%, 80–90 low-average occurring in 16.1% of the population and a score of 90–110 is likely to be found in 50% of the population. Score of 110–120 is high average (16.1%), 120–130 (6.7%) is superior and 130 (2.2%) is very superior. The WASI-II has good (.87) to excellent (.91) internal consistency, excellent (.95) test-retest stability and excellent inter-rater reliability (.94–.95) with a child sample (Wechsler 2011).

#### Incidents & Diagnosis

Information on the number of incidents a person has been involved in are split into two categories: major and minor. A major incident refers to any time physical management has to be used, for example if a young person becomes aggressive and a restraint is used, or if there is a risk to life, for example, a serious self-harm attempt. A minor incident refers to other events that may require intervention but where there is no need for physical management, for example, if a young person makes inappropriate comments to another young person or member of staff, or if there is a self-harming incident that is not deemed to be life threatening. Diagnosis received prior to entering or whilst in residence was divided in to Autism Spectrum Disorder, Attention Deficit Hyperactivity Disorder, Post-traumatic Stress Disorder and Other.

#### Type of Crime & Length of Stay

Although some young people enter the secure children’s hospitals on a welfare order, they too may have a history of offending. Having committed a crime was defined by there being police involvement and a charge being given. When the young people had a history of offending, the type of crime they had committed was recorded. For those on a youth custodial sentence order, the crime that led to them being sent to a secure children’s home was recorded, whereas for the young people on a welfare order, the most serious crime they had committed was recorded. Once again, the types of crimes that the young people had committed were categorised for ease of analysis - acquisitive offending, sexual offending, weapon related offending violent offending and other offending. This was measured in weeks by seeking out the young person’s admission date and the date of their final review meeting, which is held before a young person leaves the secure children’s home.

### Ethics

Ethical approval was granted from Cardiff Metropolitan University’s Department of Sport and Health Sciences (approval reference number: PGT-1262). Permission was also obtained from the clinical psychologist at the secure children’s home to conduct the research in this setting. Using file information to collect data regarding the young people’s experiences was thought to be the best method, as this would avoid possible re-traumatisation of the young people as a result of having to discuss their past experiences with the researcher.

### Procedure

Data was collected by combining information from both the hard files that the young people entered the secure children’s home with between 2016 and 2018. These files included details of the young people’s family history, whether there had been any previous involvement with social services and specialist services, such as mental health teams, or crimes the young people had committed, if any. Further data was gained from computerised files from the secure children’s home database which included details of any assessments undertaken with the psychologist whilst in residence, the order they entered the secure children’s home under and how long their order was for. Information regarding the type and number of adverse childhood experiences the young people had been exposed to, scores on the TSCC, performance and verbal IQ scores as well as their full scale IQ scores obtained using the WASI-II, any diagnoses the young people may have, any crimes they have committed, the number of major and minor incidents they’ve had whilst in residence and finally the length of their stay at the secure children’s home was collated.

## Results

Due to the nature of the secure children’s home and the differing lengths of sentences given to the young people, some data was missing. A series of multiple imputations were carried out to calculate values for this missing data, see TheRMUoHP Biostatisics Resource Channel (2013) for an example. When the analyses indicated that there was too much missing data the cases were excluded from the analysis (*n* = 17) leaving 60 in the final cohort.

### ACEs

The frequencies of the adverse childhood experiences identified by Felitti et al. (1998) were calculated and are presented in Table 1. Within the sample, participants were most likely to report having been exposed to between three and six ACEs, making up 63% of the sample’s total ACE score. The mean number of total ACEs was 4.20 (*SD* = 2.081). First, a Pearson’s *r* correlational analysis was run in order to determine if there were any associations between exposure to one type of ACE and likelihood of being exposed to further ACEs. Table 2 provides information regarding correlations between the 9 different categories of ACEs.

**Table 1:** Frequencies of individual ACEs identified in the current sample reported as percentages

	Present (%)	Not Present (%)
Parental separation Verbal abuse Domestic violence Neglect Parental substance abuse	80 66.7 58.3 50 50	20 33.3 41.7 50 50
Sexual abuse	38.3	61.7
Physical abuse	35	65
Parental mental health problems	33.3	66.7
Parental incarceration	13.3	86.7

**Table 2:** Correlation matrix showing correlations between the different types of ACEs

	Verbal abuse	Sexual abuse	Physical abuse	Neglect	Parental substance abuse	Domestic violence	Parental incarceration	Parental separation	Parental mental health	Total ACEs
Verbal abuse		.048	.222	.495**	.283*	.693**	.277*	.000	.350**	.754**
Sexual abuse	–		.212	.103	.240	.041	−.007	−.034	.024	.405**
Physical abuse	–	–		.314*	.105	.053	.226	−.070	.148	.505**
Neglect	–	–	–		.267*	.304*	.294*	−.167	.141	.598**
Parental substance abuse	–	–	–	–		−.034	.392**	−.250	.212	.501**
Domestic violence	–	–	–	–	–		.033	.085	.167	.524**
Parental incarceration	–	–	–	–	–	–		−.049	−.069	.437**
Parental separation	–	–		–	–	–	–		.000	.149
Parental mental health	–	–	–	–	–	–	–	–		.445**
Total ACEs	–	–	–	–	–	–	–	–	–	

*p < .05; ** *p* < .001

**Fig. 1:**
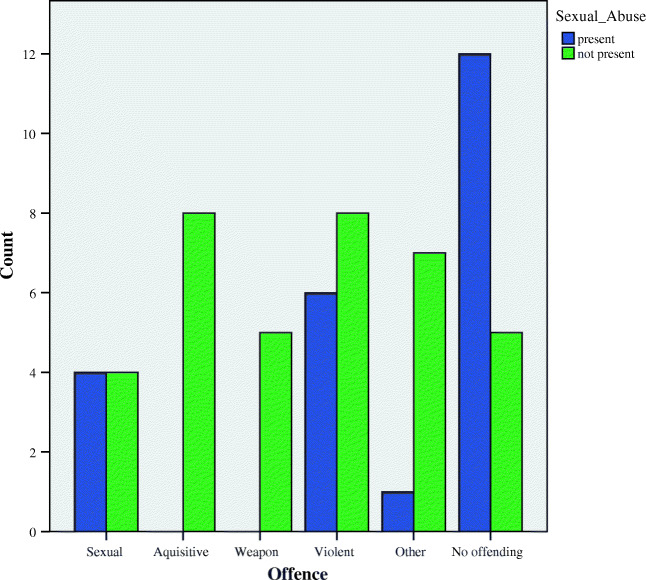
Types of offences committed by those with and without histories of sexual abuse

Next, correlational analyses were used to investigate if there was a relationship between any of the 9 ACEs and other variables thought to represent emotional and behavioural dysfunction. A number of significant correlations were found. Verbal abuse was found to be significantly negatively correlated with sexual preoccupation (*r*(58) = −.277, *p* = .032) and positively correlated with sexually harmful behaviour (*r*(58) = .296, *p* = .021). Significant positive correlations were also observed between exposure to sexual abuse and the number of minor incidents young people had been involved in (*r*(58) = .308, *p* = .017), exposure to child sexual exploitation (*r*(58) = .436, *p* < .001), displays of sexually harmful behaviour (*r*(58) = .356, *p* = 0.05) and the offence a young person committed (*r*(58) = .276, *p* = .033). Further analyses revealed that of the 38% who had been exposed to sexual abuse, the majority did not commit any type of offence (Fig. 1), whilst those that had committed a crime were most likely to commit an act of violence.

Analyses revealed significant positive correlations between exposures to parental incarceration and elevated scores on TSCC scales relating to depression (*r*(58) = .376, *p* = .003) and dissociation fantasy (*r*(58) = .303, *p* = .019). Significant positive correlations were also found between exposure to physical abuse during childhood and displaying sexually harmful behaviour (*r*(58) = .341, *p* = .008) and between exposure to domestic violence and displays of sexually harmful behaviour (*r*(58) = .266, *p* = .04).

Finally, significant negative correlations were found between being exposed to parental substance abuse and elevated scores on the sexual preoccupation scale of the TSCC (*r*(58) = −.262, *p* = .043) and between exposure to parental mental health problems and performance IQ scores (*r*(58) *=* −.285, *p* = .027). However, correlations between exposure to different types of ACEs and likelihood of experiencing the various negative outcomes identified in the research were weak, with only 7–14% of the variance being explained.

### Substance Abuse

It was found that of the 60 young people included in the study, 87% had engaged in substance use prior to entering the secure children’s home. A significant negative correlation was observed between young people’s experiences of substance abuse and the length of time in the secure children’s home (*r*(58) = −.497, *p* < .001) and a significant negative correlation between substance abuse and displays of sexually harmful behaviour (*r*(58) = −.329, *p* = .010).

### Child Sexual Exploitation and Sexually Harmful Behaviour

When looking at incidents of child sexual exploitation and sexually harmful behaviour within the sample, it was found that prior to entering the secure children’s home, 38% of the young people had been exposed to child sexual exploitation, whilst 35% of young people had displayed sexually harmful behaviour.

**Fig. 2:**
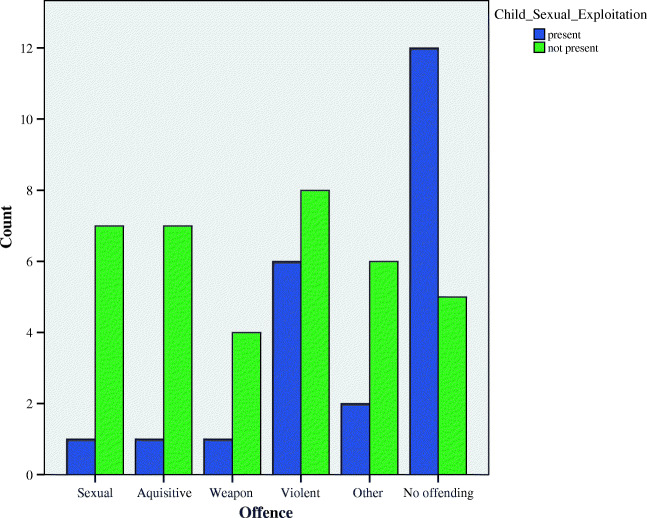
Types of offences committed by those with and without exposure of child sexual exploitation

A number of significant correlations were found between exposure to child sexual exploitation and negative outcomes, for example, there appeared to be a significant positive relationship between child sexual exploitation and young people’s scores on the sexual concerns scale of the TSCC (*r*(58) = .264, *p* = .042) and child sexual exploitation and offences committed by the young people (*r*(58) = .413, *p* < .001), specifically, those that had been exposed to child sexual exploitation were not likely to have committed any offences (Fig. 2).

Analyses were also conducted to determine if there was a relationship between displays of sexually harmful behaviour and behavioural dysfunction. Significant positive correlations were observed between displays of sexually harmful behaviour and performance IQ (*r*(58) = .280, *p* = .03), number of minor (*r*(58) = .289, *p* = .025) and major incidents (*r*(58) = .259, *p* = .046), and their length of stay (*r*(58) = .276, *p* = .033).

### IQ Scores

Mean scores for verbal and performance IQ scales, as well as full scale IQ scores are presented in Table 3. With regard to correlational analyses investigating the relationships between scores on the IQ scales and other variables, it was found that young people’s verbal IQ (*r*(58) *=* −.357, *p* = .005), performance IQ (*r*(58) = −.354, *p* = .005) and full scale IQ (*r*(58) = −.388, *p* = .002) scales were all significantly negatively correlated with scores on the anger scale of the TSCC. A significant negative correlation was also found between full scale IQ scores and number of major incidents that a person had been involved in whilst at the secure children’s home (*r*(58) = −.268, *p* = .038).

**Table 3:** Mean scores and standard deviations for IQ scores across three scales

	Mean	SD
Verbal IQ	84.39	15.62
Performance IQ	86.01	13.67
Full Scale IQ	83.61	14.35

The average range of IQ is 85–115

### Trauma Symptom Checklist

Mean scores for the 10 items on the trauma symptom checklist are reported in Table 4. A score of 65+ is deemed clinically significant for the anxiety, depression, anger, PTSD and dissociation scales, whereas a score of 70+ is thought to reach clinical significance for the sexual scales. Correlational analyses focusing on individual items on the TSCC revealed significant positive correlations between scores on the anger scale and the number of minor (*r*(58) = .315, *p* = .014) and major incidents (*r*(58) = .362, *p* = .005) that a young person had been involved in. A significant negative correlation was also identified between anger scores and diagnoses of mental health problems (*r*(58) = −.284, *p* = .028). Furthermore, a significant positive correlation was seen between young people’s scores on the dissociation overt scale of the TSCC and their length of stay at the secure children’s home (*r*(58) = .258, *p* = .046), whilst a significant negative correlation was found between scores on the dissociation fantasy scale and young people’s diagnoses (*r*(58) = −.259, *p* = .045).

**Table 4:** Mean and standard deviation scores for each of the 10 items on the trauma symptom checklist

	Mean	SD
Anxiety	50	12.17
Depression	51.38	13.56
Anger	52.13	13.03
PTSD	52.62	12.21
Dissociation	52	11.51
Dissociation overt	53.47	11.83
Dissociation fantasy	46.64	9.27
Sexual concerns	52.03	15.33
Sexual preoccupation	51.19	16.59
Sexual distress	52.12	18.89

### Diagnosis & Offence

When looking at offence types within the sample, the majority of young people in the secure home (48%) did not have a mental health diagnosis. However, amongst those with a mental health diagnosis, the most commonly reported in this setting was ADHD (58%). With regard to offending behaviour, the majority of the young people had not committed an offence prior to entering the secure children’s home (28%). Out of the 43 young people who had an offending history, 37% engaged in some kind of violent offending.

### Incidents & Length of Stay

Data regarding the number of major and minor incidents that young people had been involved in during their time in the secure children’s home was collected. The mean number of minor incidents that the young people in the population were involved in was 9.12 (*SD* = 11.05), whilst the mean number of major incident involvement was 13.9 (*SD* = 15.95). There was a significant positive correlation between the number of minor incidents a person was involved in the secure children’s home and the number of major incidents they were involved in (*r*(58) = .831, *p* < .001) and their length of stay (*r*(58) = .440, *p* < .001). There was also a significant positive relationship between the number of major incidents and the length of time spent in the secure children’s home (*r(58)* = .343, *p* = .007). With regard to how long the young people were accommodated in the secure children’s home for, the shortest amount of time spent was eight weeks, whilst the longest stay was 108 weeks (mean = 25.27 weeks, *SD* = 20.1).

### Regression Models

A series of multiple regressions were completed to investigate which variables best predicted the criterion variables minor incidents, and major incidents and length of stay. A significant model (*F*_3,59_ = 4.614, *p* < .0005) was observed. The model explained 20% of the variance in minor incident involvement by young people (Adjusted R^2^ = .198). Table 5 provides information about regression coefficients for the predictor variables entered into the model. Scores on the anger scale of the TSCC were a significant predictor, with a positive relationship to number of minor incidents. Sexual abuse and sexually harmful behaviour were not significant predictors.

**Table 5:** The unstandardized and standardised regression coefficients for the variables entered into the model as predictors of minor incidents

Variable	B	SE B	***β***	p
Sexual abuse	3.642	2.984	.162	.227
Sexually harmful behaviour	5.102	2.946	.222	.089
Anger	.227	.105	.267	.035

A multiple regression was also performed in order to determine the contributions of predictor variables to ‘major incidents’. The regression model was a significant predictor for 24% of the variance (Adjusted R^2^ = .236) of number of major incidents: *F*_3,59_ = 5.777, *p* < .005. Table 6 provides information regarding regression coefficients for the predictor variables entered into the model. Displays of sexually harmful behaviour and anger were significant predictors and had a positive relationship with major incidents, whilst full scale IQ was not a significant predictor.

**Table 6:** Standardised and unstandardized regression coefficients for variables entered into the model as predictors of major incidents

Variable	B	SE B	***β***	p
Sexually harmful behaviour	10.109	4.020	.305	.015
Anger	.317	.157	.259	.048
FSIQ	−.265	.146	−.238	.075

Finally, a multiple regression was used to investigate the contributions of predictor variables to ‘length of stay’. A significant model emerged (*F*_5, 59_ = 6.975, *p* < 0.001), accounting for 39% of the variance in the young people’s length of stay (Adjusted R^2^ = .392). Information regarding regression coefficients for the predictor variables can be found in Table 7. Substance abuse was the only variable found to be a significant predictor, with a negative correlation with length of stay. Number of minor and major incidents, sexually harmful behaviour and anger were not significant predictors of length of stay.

**Table 7:** Standardised and unstandardized regression coefficients for variables entered into the model

Variable	B	SE B	***β***	p
Minor incident	.646	.353	.355	.073
Major incident	.036	.248	.028	.887
Substance abuse	−26.058	6.711	−.445	< .001
Sexually harmful behaviour	.833	4.908	.020	.866
Anger	.001	.177	.001	.996

## Discussion

The aim of the present study was to investigate what factors are predictive of emotional and behavioural dysfunction in adolescence by using file information from young people resident in a secure children’s home. When considering what factors are predictive of behavioural and emotional dysfunction in adolescence, regression models found that minor incidents, described as incidents of non-life threatening self-harm or non-physical displays of aggression, appeared to be predicted by elevated scores in the anger scale of the TSCC. These findings may be explained by a study conducted by Klonsky (2009) who investigated the function of self-harm in young adults. Klonsky (2009) found that the primary reason endorsed for engaging in self-harming behaviours was related to affect regulation, with 85% of participants stating that they used self-harm to release a build-up of emotions. Furthermore, participants also frequently reported that self-punishment was a reason for self-harming, for example, 69% of participants stated they used self-harming behaviours to express anger at themselves, however, this tended to be rated as a secondary reason. It may be the case that the young people in the present study felt anger at what had happened to them in the past and therefore used self-harming as a way to cope with the negative feelings associated with their childhood experiences.

The data revealed that major incidents young people had been involved in, which often involved physical restraint, were also predicted by young people’s anger ratings. This finding is in line with the evidence base in the field of neurology linking trauma, frontal lobe deficits and displays of aggression. Previous research has demonstrated that both trait anger (Buss and Perry 1992) and state anger (Harmon-Jones et al. 2004) are associated with increased left frontal lobe activity. Furthermore, Hortensius et al. (2011) demonstrated that increased activity in the left frontal lobe was associated with increased behavioural aggression in 40 female participants, measured by the duration of noise blasts given to opponents during a game. Therefore, it may be the case that ACE exposure exacerbates frontal lobe deficits and together these elements pre-dispose individuals to increased levels of aggression. Alternatively, it may be the case that as a result of the young people’s traumatic upbringings and adverse home environments where violence may be commonplace, the young people have learnt that behaving aggressively towards others is an appropriate way to express their anger and to meet their own needs (Bandura et al. 1961).

Regression analyses also found that the young people’s substance abuse was the strongest predictor of the length of time that the young people spent in the secure children’s home. Specifically, young people with the highest levels of substance abuse appeared to have the shortest stays. This may be contrary to what is expected, however, this finding may be explained by the fact that there is a zero drugs policy within the unit and thus when the young people enter the building they are detoxed from any substances they may be using or addicted to and receive regular sessions with a drugs and alcohol worker to help them understand the risks of their substance abuse and give them support to maintain abstinence from drug or alcohol abuse in the future. As the young people can be accommodated in the secure children’s home because they are deemed to be a danger to themselves, this can include substance addiction, it may be the case that once the young people have come off the substances they were using and completed work around their engagement in substance use, they are no longer deemed to be a risk to themselves and thus are seen to be fit to leave the secure environment. However, for the young people that aren’t using substances before entering the secure children’s home, it is likely that they have other problems that need to be addressed before leaving. This may explain the finding that those with the highest substance use appear to spend the least amount of time in the secure children’s home.

Of the children exposed to verbal abuse, they were also significantly likely to have experienced other ACE’s including neglect, parental substance abuse, domestic violence and parental incarceration. Domestic violence and verbal abuse to the child showed the strongest correlation. This is in line with previous research, which has consistently demonstrated a relationship between domestic violence and abuse towards children, with one study reporting that approximately 40% of known child abuse victims in America also report having been exposed to domestic violence (Children’s Bureau 1999). Furthermore, principles of the Ecological Model (Bronfenbrenner 1977) propose that if a young person has a close proximal relationship to the perpetrator of the domestic violence, then this increases the likelihood of victimisation, that is, sharing a common domicile with the abuser can increase the opportunities for violent encounters. With regard to the relationship between verbal abuse and parental mental health problems, Sidebotham et al. (2016) found that amongst 175 serious case reviews regarding instances of child abuse or neglect, parental mental health problems were identified in 53% of these cases.

Previous research (Dong et al. 2003) has shown that exposure to sexual abuse during childhood is strongly associated with experiencing other ACEs, especially as the severity of the sexual abuse increases. However, this was not observed in this cohort as sexual abuse was not significantly correlated with any other type of ACE exposure. This result may be explained by an under-reporting of the young people’s sexual abuse histories as they may not have felt ready to disclose their sexual abuse to staff. Or they may have only just come into contact with social services and so their full history was incomplete. Another explanation is that sexual abuse can occur in family contexts that are absent of domestic violence, parental separation, parental substance abuse and physical violence.

Correlational analyses revealed that exposure to sexual abuse as a child was associated with the most adverse outcomes, such as being an increased risk of being exposed to child sexual exploitation (a specific form of sexual abuse) and displaying sexually harmful behaviours. Furthermore, exposure to sexual abuse appeared to predict involvement in major incidents, such as occasions where physical management on behalf of the staff is required due to incidents of physical aggression or threats to life. These findings are echoed by Green et al. (2005) who investigated the prevalence of risky sexual behaviours in women who had been exposed to trauma, verses no trauma, during adolescence. They found that women who had histories of sexual assault or abuse had an increased likelihood of engaging in risky sexual behaviours. Engaging in voluntary sex from an earlier age or having more sexual partners was higher. As was violence and suicidal ideation, especially if the women had been repeatedly exposed to abuse. Romans et al. (1995) also found a strong relationship between a history of childhood sexual abuse and incidents of self-harm later in life whereby 95% of the women in their study who reported self-harm also reported a history of sexual abuse. Whilst causal links cannot be established from this research, there were no reports of self-harm in the case histories occurring prior to the first incident of sexual abuse taking place. This suggests that for some service users it was the sexual abuse that led to the self-harm and the self-harm that led to the need for a secure children services.

### Strengths and Limitations

The main advantage of the current research is that it used case files to collect data regarding the young peoples’ historical information. This allowed for objective data collection and was not influenced by subjective interpretations or clinical opinions. At any one time, the maximum number of young people that the home can accommodate is 22. Using file information gave the opportunity to draw on a wider data base of cases. The limitation of using the young person’s case files is that the data was sometimes incomplete or are awaiting further documentation to be gathered from external agencies. As a result, the data used in the current research may not reflect the complete nature of the young peoples’ lives. It is also worth noting that issues such as sexual abuse or domestic violence are often hidden problems that are not always known to services. Additionally, when full case histories are received, they often don’t record the severity or frequency of the traumatic incidents that the young people have been exposed to, which may be an important factor. A final point to note is that although correlational research is able to identify statistical relationship between variables, it is not able to provide clarity around the causation of these patterns.

### Implications

To the researcher’s knowledge this is the largest data set obtained yet reported from a UK secure children’s home. The current research was the first to explore the statistical link between ACEs and the frequency of incidents that occur in adolescents in a secure children’s home. It was also the first study to statistically examine the factors that predict the length of time that young people spend in a UK secure children’s home. This research has highlighted that in the current population; exposure to ACEs can have a detrimental impact on the young people’s developmental outcomes. Specifically, it has been demonstrated that as a result of their adverse childhood, the young people experienced a greater level of anger, evidenced through elevated scales on the TSCC. These elevated anger scores have further been shown to be a predictor of the number of major and minor incidents that the young people become involved in whilst in the secure children’s home, which in turn have been found to be correlated with the length of time that the young people spend in the service. These findings highlight that it may be beneficial to intervene early with young people who have been exposed to numerous ACEs and traumatic experiences in order to reduce some of the impact that these exposures may have on them in the future, and importantly, the length of time that young people spend in secure environments.

Research by the Early Intervention Foundation (2017) has found that there is still a gap in the evidence base regarding the effectiveness of early interventions with only a quarter of interventions demonstrating evidence of improving outcomes in childhood. This research supports the rational of including whole family interventions to address issues associated with exposure to childhood adversity, such as Child Parent Psychotherapy (CPP; Lieberman et al. 2018). Public education centred around different types of adverse childhood events and the impacts these can have on a child’s development and life course is also needed (The Frameworks Institute 2015). It is hoped that if there is a greater public awareness about ACEs then young people who have been exposed to such experiences will be better understood and more easily identified by the general public as children need help rather than labelling them as ‘naughty children’.

With regard to policy implications, institutions should adopt the principles of the Trauma Recovery Model (TRM; Skuse and Matthew 2015). It proposes that prior to any offence related work taking place the underlying needs of the young person should be met, such as engaging in relationship building so they can have a secure base to trust adults and enable any disclosures if they need to be made. By doing so young people are more likely to show willingness to work through their trauma, which if addressed should in turn lead to a reduction in their risk behaviours.

### Future Research

Further research needs to determine if there is a critical period during childhood where exposure to ACEs has the biggest impact on future outcomes. It may also be interesting to address questions surrounding the frequency and severity of ACE exposure, and whether or not young people more at risk of experiencing adverse outcomes following multiple exposures to the same ACEs compared to just one singular traumatic experience. Future research may also be conducted using a larger sample than the one in the present study, which would allow for separate analyses between different sub groups, such as males compared to females or young people on a welfare order compared to those on a youth custody service order. This may be achieved by expanding the length of time data is collected from and/or by conducting research across a number of different sites within the UK.

In conclusion, the present research aimed to be the first UK study to investigate what factors are statistically predictive of emotional and behavioural dysfunction. The results indicate that exposure to one adverse experience in childhood is correlated with further exposure to adverse experiences, especially if the young people had experienced verbal abuse. Furthermore, exposure to sexual abuse appeared to be most strongly associated with behavioural dysfunction, such as involvement in more incidents and displays of sexually harmful behaviours. With regard to the length of stay, the young people’s substance used appeared to be the greatest predictor. Although the present study adds to the growing literature base on the effects of ACE exposure on outcomes in adolescence, further research still needs to be conducted to enhance the understanding of how to reduce the number of incidents and the length of time that young people spend in secure settings using a nationwide sample obtained from numerous UK secure children’s homes.
